# Fast and reliable advanced two-step pore-size analysis of biomimetic 3D extracellular matrix scaffolds

**DOI:** 10.1038/s41598-019-44764-5

**Published:** 2019-06-07

**Authors:** Tony Fischer, Alexander Hayn, Claudia Tanja Mierke

**Affiliations:** University of Leipzig, Faculty of Physics and Earth Science, Peter Debye Institute of Soft Matter Physics, Biological Physics Division, Linnéstr. 5, 04103 Leipzig, Germany

**Keywords:** Cellular motility, Biological physics

## Abstract

The tissue microenvironment is a major contributor to cellular functions, such as cell adhesion, migration and invasion. A critical physical parameter for determining the effect of the microenvironment on cellular functions is the average pore-size of filamentous scaffolds, such as 3D collagen fiber matrices, which are assembled by the polymerization of biopolymers. The scaffolds of these matrices can be analyzed easily by using state-of-the-art laser scanning confocal imaging. However, the generation of a quantitative estimate of the pore-size in a 3D microenvironment is not trivial. In this study, we present a reliable and fast analytical method, which relies on a two-step 3D pore-size analysis utilizing several state-of-the-art image analysis methods, such as total variation (TV) denoising and adaptive local thresholds, and another crucial parameter, such as pore-coverage. We propose an iterative approach of pore-size analysis to determine even the smallest and obscure pores in a collagen scaffold. Additionally, we propose a novel parameter, the pseudo-pore-size, which describes a virtual scaffold porosity. In order to validate the advanced two-step pore-size analysis different types of artificial collagens, such as a rat and bovine mixture with two different collagen concentrations have been utilized. Additionally, we compare a traditional approach with our method using an artificially generated network with predefined pore-size distributions. Indeed, our analytical method provides a precise, fast and parameter-free, user-independent and automatic analysis of 3D pore topology, such as pore-sizes and pore-coverage. Additionally, we are able to determine non-physiological network topologies by taking the pore-coverage as a goodness-of-fit parameter.

## Introduction

Filamentous networks play a role on various length scales that range from the interior cell level based cytoskeletal networks, over extracellular matrix based microenvironmental networks to organ-scale networks, such as vascular networks that are subject to blood coagulation. These networks are commonly formed by the polymerization of biomacromolecules, such as monomeric proteins, and assemble to three-dimensional (3D) structures. The physical properties, such as the pore-size, fiber diameter and fiber length determine the matrix mechanical properties and modulate cellular or tissue functions. In more detail, the pore-size of these matrices has been shown to broadly affect cellular function, such as cell adhesion and polarization, and hence requires to be determined precisely^1–6^. Moreover, the knowledge of the distribution of the pore-sizes of these biopolymer architectures, seems to be crucial for the analysis of the mechanical properties, diffusive processes and the migration of cells through these networks. As the analysis of these matrix properties is important, a broad variety of techniques have been developed and further improved, including microscopy^7–9^ and scattering methods^10–12^, rheological and transport approaches^8,13,14^.

The average pore- or mesh-size is one of the most important parameters to physically characterize the structure of biopolymeric filamentous networks, as it determines both the rheological phenotype and the diffusivity of these filamentous networks^8,13,14^. Due to the convoluted information of confocal microscopic images and the randomness of biological networks, the extraction of physical parameters is not trivial and hence not a straightforward approach.

The current pore-size analysis methods do not appropriately address the user independent analysis of these important structural parameters and the classical bubble analysis ignores the residual fluid volume that is not further determined.

Hence, in order to address these concerns, we used the well-established 3D bubble analysis algorithm^15^ in order to analyze large cube sizes of confocal stack images and thereby implemented a 3D Euclidian distance map (EDM) based bubble analysis^16^. Moreover, to account the altered absorption and refraction due to the image stack total height, a per plane TV based denoising^17^ can be performed and a local adaptive threshold can be set. In addition, a morphological closing can be used to remove rarely occurring, small, not connected objects and artifacts, which represent no obstacle for the migration of cells, such as cancer cells or fibroblasts, through connective tissues.

## Materials and Methods

### Collagen I matrices

We used *in vitro* polymerized 3D extracellular matrices as published previously^18–20^. A phosphate buffered solution with pH 7.4 (ionic strength of 0.7, 200 mM phosphate) consisting of a mixture of sodium dihydrogen phosphate (Sigma Aldrich, Cat.No: 71507) and disodium hydrogen phosphate (Sigma Aldrich, Cat.No: 71636) in ultrapure water was mixed with collagen type I monomers extracted from rat tail (Serva, Heidelberg, Germany, Cat.No.: 47256) and calf skin (Biochrom, Berlin, Germany, Cat.No.: L7213) with a mass fraction of 1:2, respectively, and kept on ice (4 °C). To fixate 3D collagen scaffolds for imaging, glass coverslips of 13 mm diameter were functionalized by coating with (3-Aminopropyl)trimethoxysilane (APTMS) (Sigma Aldrich, Cat.No: 281778). 100 µl collagen-buffer solution was transferred onto APTMS-coated coverslips and polymerized in an incubator at 37 °C, 95% humidity for 2 hours. Subsequently, polymerized matrices were washed 3 times with phosphate buffered saline (PBS) and kept hydrated in an incubator at 37 °C, 95% humidity.

### Imaging of 3D collagen I matrices

**Figure 1:**
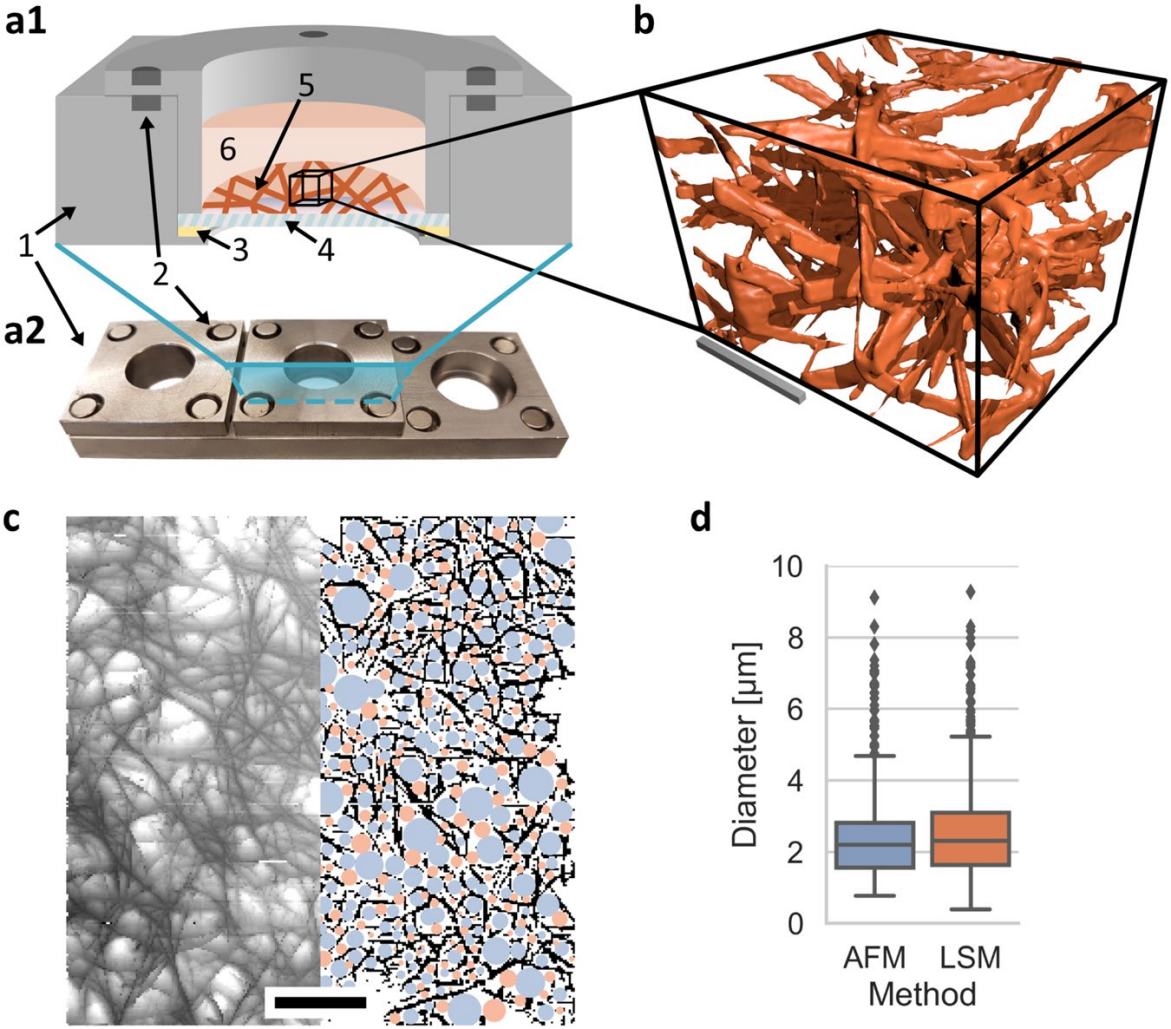
Illustrations of sample mounting and final recording. (**a1**) An illustration of a prepared sample is presented: 1 metal frame, 2 fixating magnets, 3 petrolatum sealant, 4 glass coverslip, 5 collagen matrix, 6 PBS. (**a2**) Photography of a crafted mounting device with three mounting pots. (**b**) 3D visualization of a smaller section of an image cube of the TAMRA-labelled collagen scaffold is provided. The scale bar is 20 µm. (**c**) A half-width blend of the grey-value image of a collagen network surface height-map (left) using AFM and the resulting segmentation and detected pores (right) are presented. The scale bar is 20 µm. (**d**) Both methods provide almost identical results.

3D collagen matrices were fluorescently stained overnight using 20 µg/ml 5(6)-Carboxytetramethylrhodamine N-succinimidylester (TAMRA-SE) (Sigma Aldrich, Cat.No: 21955), washed 3 times with PBS and kept hydrated. The stained gels were imaged using a CLSM (Leica TCS SP8, Mannheim, Germany) with a 40x NA/1.10 water immersion objective. To maximize image quality and keep the gels hydrated, coverslip samples were mounted in a custom-built mounting device (Fig. 1a). Images with a resolution of 2048 × 2048 pixels and vertical stack size of 600 images resulting in an image cube with edge length of 150 µm were recorded. A deconvolution was applied using the Huygens Essentials v16.10 software (Scientific Volume Imaging B.V., Hilversum, Netherlands). The full resolution images are only used for deconvolution and stored for reference and visualization. However, for pore-size determination it is sufficient to resample the recorded images. This is done so that the x-y resolution matches roughly the stack size, e.g. 600 × 600 pixels. To test whether this imaging method is sufficient to represent the actual collagen fibers, we used an Atomic Force Microscope (AFM) to record a height-map of the surface of a representative collagen network (see Fig. 1c), as well as the fluorescently stained network at the surface using the method described above (Fig. 1d).

### Porosity

Collagen scaffolds can be seen as a porous material that contains a multitude of differently shaped pores. Thus, porosity can be used as a standard measure^21^. Porosity *ϕ* is given in Eq. (1):

1 $$\varphi =\frac{{V}_{fluid}}{{V}_{cube}}$$

with fluid volume *V*_*fluid*_ and cube volume *V*_*cube*_.

### 3D Pore-size analysis

The critical metadata of an image stack, such as voxel size, were directly obtained using a custom software library written in Python utilizing the Bio-Formats library^22^. In order to deal with the scattering and absorption due to very high sample heights and thus large travel length of exciting and emitted light, an image segmentation of fibril and non-fibril volumes was performed using a per-image-plane approach. Prior to a segmentation, a total variation denoising^17,23^ was applied. Subsequently, an adaptive local threshold was used to precisely identify fibrils of brighter collagen nodes and areas of sparse scaffold. For each pixel at coordinates (*x*,*y*) in the source image *im*_*z*_ with *z* being the height in the image stack *im*, a segmentation *sgm*_*z*_ of the same size of *im*_*z*_ was calculated as given in Eq. (2):

2 $$sg{m}_{z}(x,y)=\{\begin{array}{c}1\,{\rm{if}}\,i{m}_{z}(x,y) > g(x,y)\\ 0\,\,otherwise\end{array}$$

where *g* is the cross-correlation of *im*_*z*_ with a gaussian kernel with *σ* = *k*/6, and *k* is the block-size of an equivalent window around (*x*,*y*)^23^. The 3D segmentation result *sgm* contains the *sgm*_*z*_ for each height within the image stack *im*. Subsequently, morphological closing to refine the 3D binary image *sgm* is performed. The segmentation result *sgm* can solely have the values 1 and 0, when a fibril or no fibril is present, respectively. As shown in^16^, an Euclidean Distance Transform (EDT)^24^ is applied to the non-fibril part of *sgm*. The Euclidean Distance Map (*EDM*) represents the shortest distance of each non-fibril pixel to the neighboring collagen fibrils. After smoothing of the *EDM* with a gaussian kernel, local maxima are determined. These maxima and corresponding values of the original, not smoothed *EDM* represent coordinates and radii of the largest bubbles (spheres) that would fit in each respective 3D pore of the collagen scaffold. The actual pore size *ζ* is given by the median of all pore diameters *d*_*p*_ of an individual collagen scaffold sample, as seen in Eq. (3):

3 $$\zeta ={\rm{median}}({d}_{p})$$

### Determination of the residual fluid volume

So far, our model fits spherical objects into the spaces between the fibrils, the so-called fluid-phase, to determine the available space for the migration of cells. However, depending on the inhomogeneous structure of a biopolymer network, the fluid-phase is not completely covered by spherical pores. In Eq. (4), the residual volume *V*_*residual*_ is the difference of fluid-phase volume *V*_*fluid*_ and the volume *V*_*pores*_ that all currently detected pores occupy, when using a simple pore-size analysis approach:

4 $${V}_{residual}={V}_{fluid}-{V}_{pores}$$

Therefore, we calculate the actual volume of the non-fibril space or fluid-phase ${V}_{fluid}={\sum }^{}sg{m}_{fluid}(x,y,z)\ast c$ with *sgm*_*fluid*_(*x*,*y*,*z*) being each non-fibril voxel multiplied with the voxel-to-micron conversion factor *c*. The pore bubbles occupy the actual pore volume *V*_*pores*_. Overlaps are considered and hence only count once. This is achieved, when we draw a ball with a value of 1 at each position with respective radii in a zero-image for each detected pore-bubble, termed *sgm*_*pores*_, and measure the ball’s occupied volume. Thus, the *V*_*residual*_ is a measure for the fluid-phase space that is not covered by the previously fitted pore-bubbles. In general, the more fluid volume is covered by detected bubbles, the smaller is the amount of *V*_*residual*_. Moreover, when the scaffold contains only spherical holes, they can be completely covered by spherical bubbles, and hence *V*_*residual*_ is zero.

### Calculation of the pseudo pore diameter

In order to address the inhomogeneity of a certain structure, when *V*_*residual*_ > 0, we determine a virtual pore diameter, termed pseudo pore diameter *ξ*_*pseudo*_. Therefore, all pores are assumed to be spherical and thus we can calculate it, as given in Eq. (5):

5 $${\xi }_{pseudo}=\sqrt[3]{\frac{6\ast {V}_{fluid}}{{n}_{pores}}}$$

where *V*_*fluid*_ is the fluid volume and *n*_*pores*_ represents the number of pores. In summary, the resulting pseudo pore diameter *ξ*_*pseudo*_ is the mean pore diameter of a virtual fluid volume with completely spherical pores.

### Detection of residual fluid pores

To improve our model and fit more pore bubbles inside the fluid phase, we propose a novel method termed residual fluid pore detection. After detecting the 3D pore size as explained above, we carry out the pore detection algorithm iteratively on the residual fluid volume *V*_*residual*_. This results in a new residual *edm*_*residual*_ with new detected pore diameters *d*_*residual*_. Hence, the resulting pore size is determined in Eq. (6) as follows:

6 $${\zeta }_{residual}={\rm{median}}({d}_{pores}\cup {d}_{residual})$$

The main advantage of the approach is that our algorithm now detects new pores separately in shallow space along larger pores without over-fitting large spaces.

### Network simulation

In order to test our advanced method, a comparison with traditional methods is eligible. Therefore, we developed an algorithm that mimics a simplified biopolymer network with predefined pore distributions. Firstly, we generate a random normal distribution of pore radii. We then use a custom-built packing algorithm based on Euclidean distance that draws binarized pore circles or spheres, for 2D and 3D respectively, into a zero-matrix. This results in a densely packed binary matrix with randomly sized and placed pores. Subsequently, a skeletonization algorithm^25,26^ is applied to the non-pore areas, leading to an artificial network based on predefined pore sizes. This artificial scaffold already represents a binary segmentation. Finally, we apply both a traditional method and our advanced method to this segmentation, determine the pore-size distributions and compare them to the original distribution.

### Fibroblast actin networks

Mouse embryonic fibroblasts (MEFs) were kindly provided by Dr. Fässler and cultured in Dulbecco’s modified Eagle’s medium with high-glucose (4.5 g/liter) containing 10% fetal calf serum and low endotoxin levels (<0.1 EU/ml), 2 mM L-glutamine and 100 units/ml penicillin-streptomycin (Biochrom, Berlin, Germany). 80%-confluent cells were used in passages 6–30. Cells were harvested using 0.125% trypsin/EDTA solution (<1% dead cells). We seeded MEFs on laminin coated glass cover-slips, fixed with paraformaldehyde and stained actin using Alexa Fluor 546 Phalloidin. Subsequently, the bundled actin networks in the cytoskeleton were imaged using a 63x objective in 2048 × 2048 pixel resolution in a single image plane. Finally, we segmented the actin bundle network into a binary image and applied a 2D implementation of our residual pore detection algorithm.

### Statistical analysis

The data were presented as mean or median values ± SD as indicated. Histograms are presented as univariate kernel density estimates with gaussian kernel shape. Statistical significance was analyzed using the Mann-Whitney U test with standard significance levels of 5%, 1% and 0.1%.

## Results

### Parameter-free binarization

**Figure 2:**
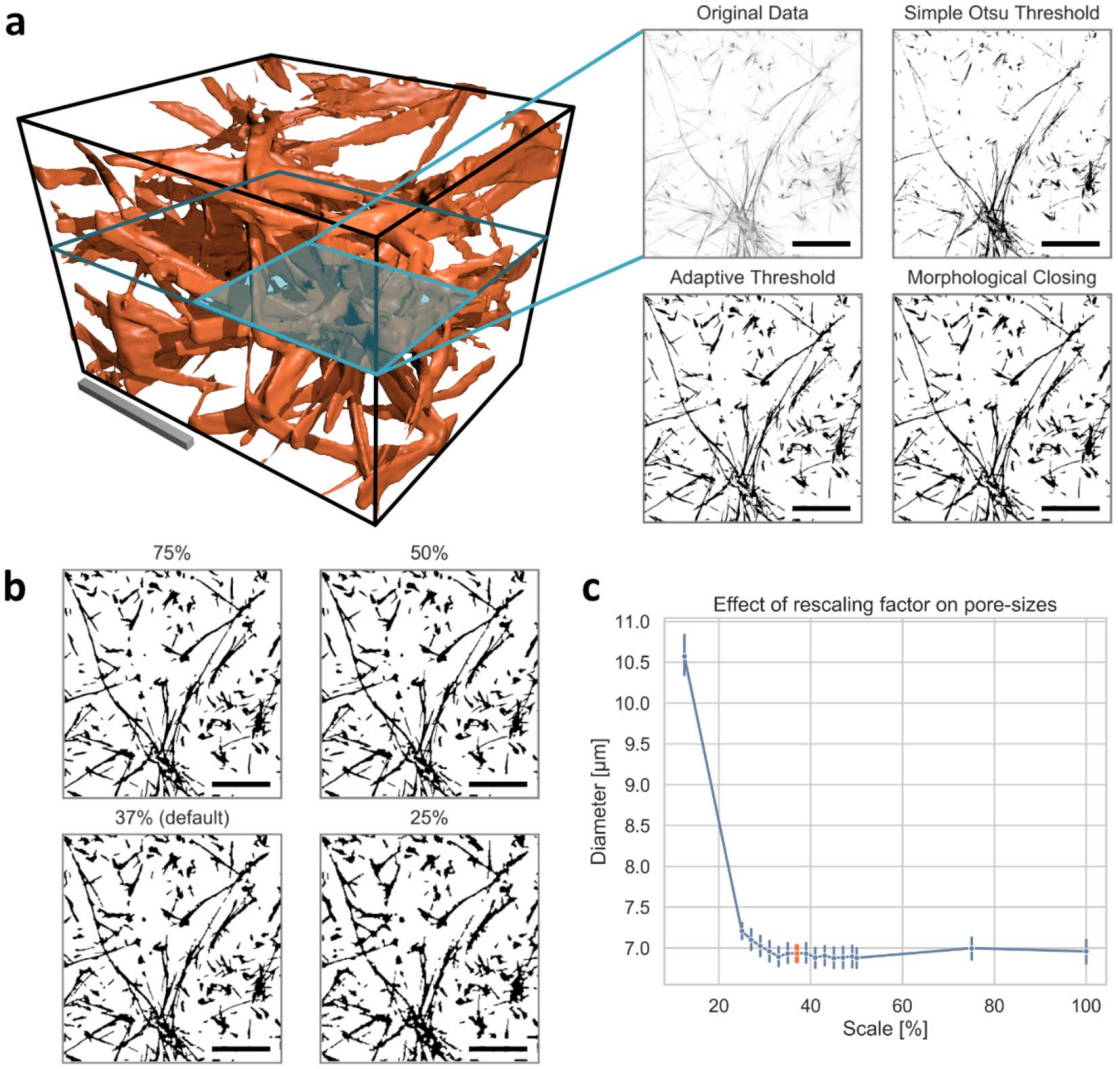
3D collagen network binarization method. (**a**) Left image: Representative image cube of a TAMRA-labelled collagen scaffold. Top left image: A representative 2D slice of original image data is shown. Top right image: A common approach using best threshold based on Otsu’s method^28^ is provided. Bottom left image: An adaptive local threshold of a denoised image is presented. Bottom right image: The final segmentation result is shown. (**b**) The effect of down-sampling of the x-y resolution on the segmentation is presented for selected percentages. (**c**) Effect of rescaling on the pore-sizes, selected 37% highlighted in orange. All scale bars are 20 µm.

Our novel approach of the segmentation is well suited to identify fibrils in 3D biopolymer networks, as seen in Fig. 2. All parameters are set automatically based on image size, illumination, contrast and various other data, and hence reveals a parameter- and thus user-independent fibril segmentation. However, the rescaling factor is a priori arbitrary and thus still user-dependent. Thus, we determined the segmentation sufficiency for our segmentation procedure and visualized the determination of the rescaling factor.

3D images are recorded with a x-y resolution of 2048 × 2048 pixels in order to maximize deconvolution efficiency. However, to minimize the calculation time and computational resource usage, we determined that it is sufficient to rescale the deconvolved images to roughly 37% of their original size (Fig. 2b). Rescaling much smaller than 37% results in a loss of the segmentation quality. This loss can be visualized using the pore-size in dependence of the rescaling factor on an exemplary collagen network, as shown in Fig. 2c. Original images are stored for reference and 3D visualization purposes.

To determine whether our segmentation approach represents the actual collagen fibers appropriately, we first recorded the surface of an exemplary collagen network at the top surface using the imaging method described above and applied our binarization method. Subsequently, we recorded a height-map of the same sample using AFM and segmented it similarly into fibril and non-fibril areas. In the next step, we compared the detected pore-size distributions and found that both methods provide almost identical results, as seen in Fig. 1d. As the surface is probed with a pyramidal tip cantilever, fibrils occur slightly broader, resulting in a slightly shifted pore-size distribution.

### Porosity and 3D pore detection of collagen matrices

**Figure 3:**
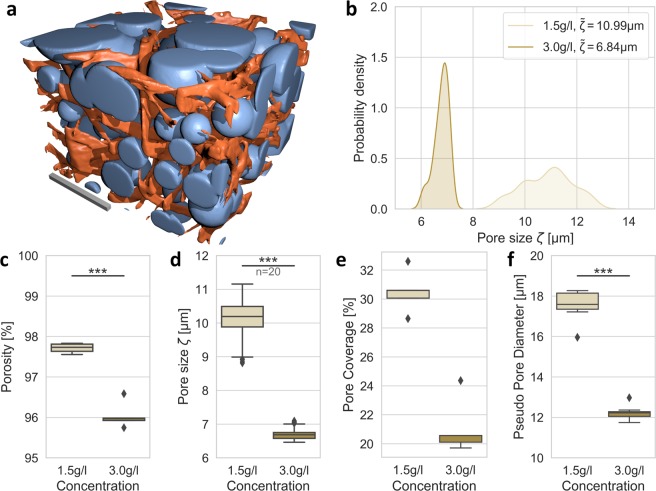
Crucial parameters to describe collagen network pore-size. (**a**) 3D visualization of detected spherical bubbles: Orange depicts segmented collagen fibers and blue represents determined bubbles. The scale bar is 20 µm. (**b**) The histogram of detected pore sizes ***ζ*** with median pore size $\tilde{\zeta }$ indicated. (**c**) The material porosity of 1.5 g/l and 3.0 g/l collagen gels is shown. (**d**) The median per image-stack provides a robust measure of pore-size ***ζ***. (**e**) The percentage of fluid volume, which is covered by detected pore spheres, is presented. (**f**) The pseudo pore diameter represents a virtual pore diameter for completely spherical fluid volumes.

An initial visualization of detected spherical bubbles for a sample 3.0 g/l collagen matrix can be seen in Fig. 3a. The detected spherical bubbles can be clearly seen and naturally overlap to a certain extent. Figure 3b shows the distribution of detected pore sizes *ζ* for each exemplary collagen concentration. A porosity of 97.89 ± 0.23% has been determined for 1.5 g/l and 95.58 ± 0.59% for 3.0 g/l collagen scaffolds which represents a relatively high material porosity for both collagen concentrations. The determined collagen porosities for two different 3D collagen matrices are shown in Fig. 3c. When taking the median pore diameter *ζ* of a single 3D image stack, we can calculate the pore-size of that respective sample. Pore-size values for 20 samples per collagen concentration are shown in Fig. 3d. We determined a median pore-size of 10.99 ± 0.95 µm for 1.5 g/l and 6.78 ± 0.27 µm for 3.0 g/l collagen matrices. However, due to the inhomogeneous structure of collagen scaffolds, spherical bubbles cover only a certain portion of the actual fluid phase. The percentage of fluid volume covered by detected spherical bubbles (termed pore coverage) is shown in Fig. 3e. Values of 32.42 ± 2.42% for 1.5 g/l and 20.44 ± 1.32% for 3.0 g/l collagen scaffolds show a decent coverage. The pseudo pore diameter was determined to be 17.54 ± 0.72 µm for 1.5 g/l and 12.19 ± 0.23 µm for 3.0 g/l networks, when completely spherical fluid volumes are assumed (see Fig. 3f).

### Residual (refined) pore detection

**Figure 4:**
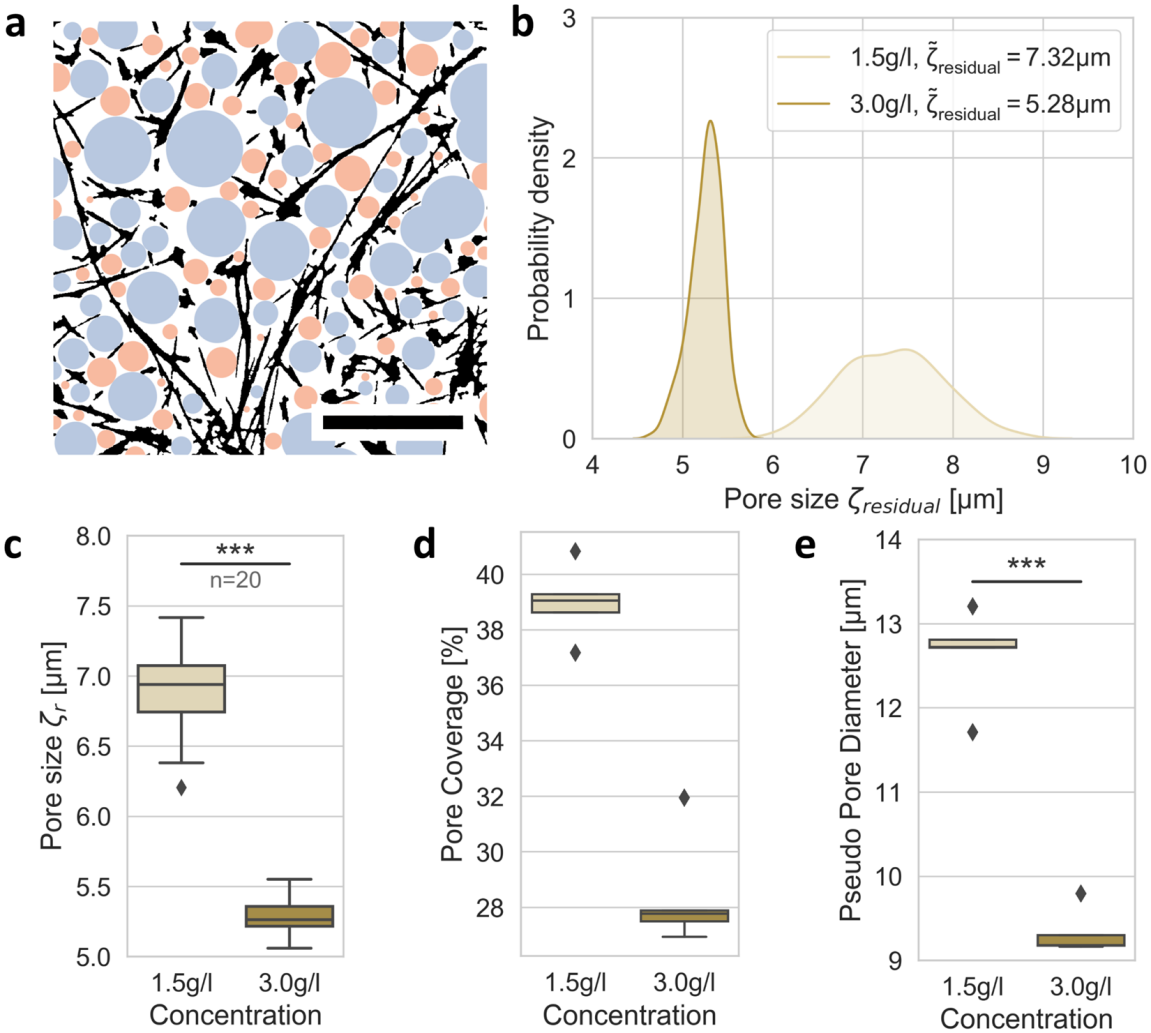
Crucial parameters with residual pore-size detection. (**a**) Visualization of detected 2D bubbles (circles) in an exemplary 2D image slice. Black depicts segmented collagen fibers, blue represents determined pores of a single analysis process, orange represents detected pores of a second residual analysis. The scale bar is 20 µm. (**b**) The histogram of detected pore diameters with median pore size $\tilde{\zeta }$_***residual***_ for the two collagen gels is shown. (**c**) The median per image-stack provides a robust measure of pore size ***ζ***_***residual***_. (**d**) The percentage of the fluid volume covered by the detected pore spheres (pore coverage) of 1.5 g/l matrices is increased compared to 3.0 g/l matrices. (**e**) The pseudo pore diameter measures a virtual pore diameter for completely spherical fluid volumes of both collagen matrices.

As stated above, we iteratively performed a second pore detection step of the fluid phase, but thereby we excluded the already detected spherical pore bubbles. In more detail, we accomplish this by taking the original network binary segmentation *sgm* and add the pore sphere binary *sgm*_*pores*_, which was determined as explained above. In the next step, we then detect pores of this new pseudo segmentation to identify auxiliary pores apart from the original ones (Fig. 4a,b).

Naturally, these pores are smaller and shift the final calculated pore-sizes towards smaller ones, as seen in Fig. 4c. Hence, these pore-sizes are 7.32 ± 0.55 µm for 1.5 g/l and 5.28 ± 0.19 µm for 3.0 g/l collagen networks. However, when using the novel two-step approach, we cover a much larger volume with the detected pore spheres, as it can be seen in Fig. 4d,e, compared to Fig. 3e. Also, the number of detected pores raises from around 1200 and 3300 for 1.5 g/l and 3.0 g/l scaffolds, respectively, to about 3200 and 7700 for both gels. These findings clearly implicate that our approach of residual pore detection is well suited to detect pores more precisely than shown in previous publications^15,16^, which cannot detect such high numbers of pores within similarly concentrated collagen matrices.

Finally, our novel approach is not restricted to only a second analysis step, but could also be iteratively carried out multiple times, detecting more and more fine detail of a biopolymer network structure. However, in this publication, we focused on only two steps to determine pore-sizes that are physiologically relevant for cell migration.

### Network simulation

**Figure 5:**
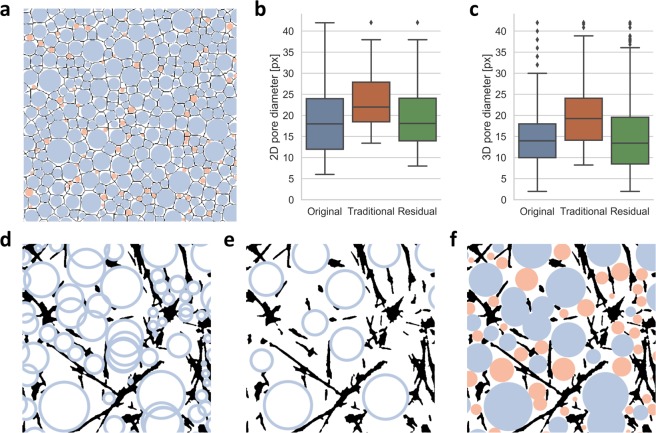
Comparison of traditional and advanced method. (**a**) Illustration of simulated 2D network. Black depicts artificial network fibril segmentation, blue represents determined pores of a single analysis process, orange represents detected pores of a second residual analysis. (**b**) Comparison of pore-sizes for a 2D artificial network. (**c**) Comparison of pore-sizes for a 3D artificial network. (**d**) With small gaussian sigma, pores overlap drastically and smaller spaces are not covered. (**e**) With large gaussian sigma, smaller pores are not detected at all. (**f**) Residual method is superior.

With knowledge of the pore-size distribution of a simulated, artificial network (Fig. 5a), we are able to compare our novel method with traditional approaches. A comparison of a traditional approach and our advanced method is shown in Fig. 5b,c both for 2D and 3D simulated networks, respectively. Indeed, our method represents the actual pore-size distributions more precisely. For an exemplary simulated 2D network with 72 pores and a pore-size of 18.83 ± 8.83 px, the traditional approach detects 47 pores with pore-size of 23.82 ± 7.48 px. Our advanced method detects 71 pores with a pore-size of 19.84 ± 8.36 px. In case of a simulated 3D network with 1120 pores and a pore-size of 14.85 ± 7.50 px, the traditional approach detects 564 pores with pore-size of 19.70 ± 6.95 px, while our advanced method detects 1127 pores with a pore-size of 14.68 ± 7.53 px. An illustration of an artificial 2D network and both 2D and 3D results are shown in Fig. 5a–f.

### Pore coverage represents a fit quality parameter

**Figure 6:**
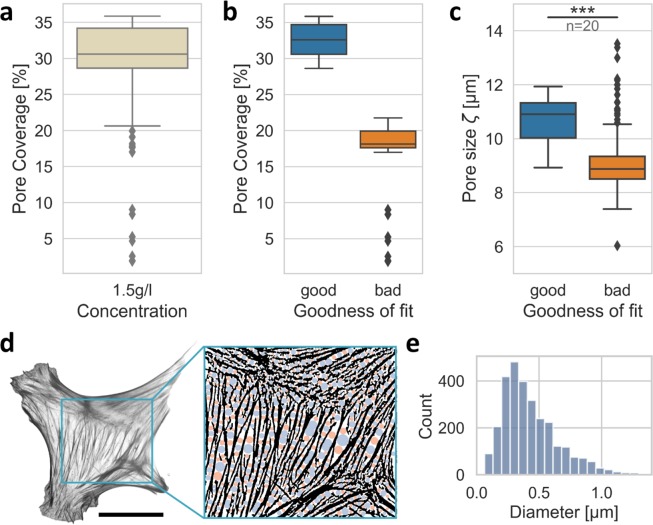
Pore coverage as a goodness-of-fit parameter for collagen networks and applicability to actin bundle networks. (**a**) The pore coverage of both normal and manipulated collagen samples taken as a single measurement. (**b**) The samples are categorized as ‘good’ (not mechanically perturbed) and ‘bad’ (mechanically perturbed) samples based on their pore coverage. (**c**) Clearly distinguishable pore-sizes of the good and bad samples show that the “flawed” (bad) samples scatter broadly and have a drastically altered pore size. (**d**) Left image: greyscale image of actin bundle network of an exemplary mouse embryonic fibroblast (MEF). Scalebar is 20 µm. Left image: illustration of segmentation and detected pores. (**e**) Histogram of actin bundle pore-sizes determined for three exemplary MEFs.

The pore coverage seems to be an excellent goodness-of-fit parameter. We hypothesized that with our advanced method it would be possible to distinguish non-physiological scaffolds. In order to test this hypothesis, we intentionally altered some collagen samples by mechanically disrupting them with a pair of tweezers. Therefore, we inserted the tips of a pair of tweezers into the polymerized network and subsequently moved the tweezer tips. In this way, the network gets permanently deformed mechanically and torn apart locally. After analyzing these mechanically perturbed collagen matrix samples (Fig. 6a), they can be clearly distinguished from non-perturbed matrices in both their altered pore coverage (Fig. 6b) and in their “flawed” pore-size (Fig. 6c).

These results indicate that the pore-coverage, especially in conjunction with our two- or possibly multi-step pore-size analysis, can be used to identify mechanically or otherwise perturbed samples of collagen fiber matrices. Moreover, the analysis may be used to detect enzymatically degraded or otherwise altered collagen matrix regions within a sample.

### Actin networks in cells

We applied a 2D implementation of our pore-detection algorithm to the actin cytoskeleton of mouse embryonic fibroblasts (MEFs), which adhered to a 10 µg/ml laminin coated glass surface for 16 hours. Indeed, the algorithm is independent of the size of the observed structure and much smaller pore-sizes compared to 3D collagen matrices can be determined, as seen in Fig. 6d,e.

## Discussion

The findings in this publication show that our novel approach of multi-step pore-size analysis is well suited to describe the pore-sizes and structure of collagen scaffolds and biopolymer networks in general. Firstly, we implemented a parameter-free and precise segmentation of fibrils, which is crucial to accurately determine scaffold properties. We have shown that the segmentation accurately depicts actual fibrils by comparing fibril segmentations of a collagen network surface obtained mechanically using AFM and fluorescence microscopy by confocal laser scanning microscopy (CLSM). Secondly, we are able not only to detect spherical pores in such a gel without significant overlaps, as discussed in^27^, but also detect smaller off-site pores that would be omitted otherwise, due to the highly inhomogeneous nature of collagen networks. This iterative process can be performed multiple times to detect even smallest pores, when they are desired.

However, we found that a second and possibly a third step is more than sufficient to cover most of the fluid phase, as the cell migrating though these collagen networks may not sense the smallest pores and hence may not alter their migration mode or migration efficiency. However, the migratory behavior of cells within these matrices needs to be investigated in more detail^5^ and functionally correlated with the nearby local advanced pore-size analysis. Moreover, we have shown that our novel analysis is highly reproduceable and leads to the same results, when repeating the experiment up to 20 times. In addition, we have demonstrated that our novel method can also be applied to quasi-static bundled actin networks in cells, such as mouse embryonic fibroblasts. In principle, other filamentous matrices, such as artificially quasi-static bundled actin networks, gelatin gels or collagen/extracellular matrix mixtures, can be analyzed using this novel approach. In fact, the algorithm is applied to pixel data. The actual pixel resolution is not relevant for the analysis. However, the imaging approach must be adapted to the size of the structures to be analyzed.

Another application field is the detection of mechanically perturbed collagen fiber matrices and possibly also of mechanically altered regions within these matrices. Hence, we have established a method to identify mechanically perturbed matrices. Moreover, cellular altered regions within the 3D collagen matrices seem to be identifiable using this novel approach.

In conclusion, we propose that our advanced multi-step pore-size analysis represents a powerful tool for the characterization of isotropic and anisotropic filamentous biological networks, such as collagen matrices by revealing a 3D pore-size distribution analysis with high coverage of the fluid phase volume. The method can be applied in future approaches to networks with morphologies different from the collagen matrices composed of mixtures to networks consisting of different types of collagen or extracellular matrix proteins.
